# Flow-Rate-Controlled Agglomeration and Ambient Oxide Phase Characteristics of Spark-Ablated Copper Nanoparticles

**DOI:** 10.3390/nano16191270

**Published:** 2026-10-08

**Authors:** Joumana El-Rifai, Eliane Bsaibess, Diaa Mereib, Corinne Bouillet, Mustapha Zaghrioui

**Affiliations:** 1 Novel Materials Development Laboratory, SAFIR (Sorbonne Abu Dhabi for Innovation and Research Institute), Sorbonne University Abu Dhabi, Abu Dhabi P.O. Box 38044, United Arab Emirates; 2 Plateforme MACLE-CVL (Microscopies, Imageries et Ressources Analytiques en Région Centre-Val de Loire), UAR 2590 (Unité d’Appui et de Recherche), 1b, Rue de la Férollerie, 45071 Orléans Cedex 2, France; 3 GREMAN (Groupe de Recherche en Matériaux, Microélectronique, Acoustique, Nanotechnologies), UMR (Unité Mixte de Recherche) 7347–CNRS (Centre National de la Recherche Scientifique), IUT de Blois, University of Tours, 15 Rue de la Chocolaterie, 41029 Blois, France

**Keywords:** spark ablation, copper nanoparticles, carrier gas flow rate, nanoparticle agglomeration, copper oxide, Raman spectroscopy, transmission electron microscopy, scanning electron microscopy

## Abstract

Spark ablation is an attractive gas-phase route for producing ligand-free metal nanoparticles, yet control over agglomeration and phase evolution during deposition remains challenging. The influence of carrier gas flow rate and deposition time on the agglomerate morphology, size distribution, and oxidation behavior of copper nanoparticles deposited onto silicon and glass substrates is investigated. Copper nanoparticles were generated by spark discharge and transported by nitrogen (N_2_) carrier gas under flow rates ranging from 0.5 to 3.5 L min^−1^ prior to deposition onto silicon substrates for 2 h and 5 h. Scanning electron microscopy reveals a transition from compact, isotropic agglomerates at low flow rates to elongated, chain-like structures at higher flow rates, indicating a shift from coalescence- and restructuring-favored conditions toward increasingly kinetically constrained aggregation. Transmission electron microscopy reveals predominantly spherical nanoparticles with a lognormal size distribution centered at approximately 10 nm, consistent with gas-phase nucleation and coalescence. Structural and spectroscopic analyses suggest CuO as the dominant oxide phase with minor Cu_2_O contributions, with Raman spectroscopy providing the primary evidence due to limitations in high-resolution TEM analysis from substrate effects and peak overlap. These findings show that carrier-gas flow rate systematically influences agglomerate structure under the investigated conditions, while oxidation may occur both during aerosol transport and following ambient exposure; the present measurements do not allow the relative contributions of these processes to be quantitatively separated.

## 1. Introduction

Copper-based nanoparticles continue to attract strong interest across heterogeneous catalysis and CO_2_/electrochemical conversion processes [1], chemical and gas sensing technologies [2], antimicrobial coatings for biomedical and environmental applications [3], and printed electronics [4], due to their high electrical conductivity, rich redox chemistry, natural abundance, and lower cost compared with noble metals. At the nanoscale, copper exhibits enhanced surface reactivity and size-dependent optical and electronic properties arising from its high surface-to-volume ratio and susceptibility to oxidation.

Copper can exist in multiple stable phases, including metallic Cu, cuprous oxide Cu_2_O, and cupric oxide CuO, which differ in oxidation state, crystal structure, electronic properties, and catalytic behavior. Cu_2_O is a p-type semiconductor with a direct band gap of approximately 2.0 eV and broad solar absorption, making it attractive for photocatalysis and solar energy conversion [5]. However, its practical performance is often limited by photocorrosion, low stability, and reduced quantum efficiency.

CuO nanoparticles possess a narrower band gap (approximately 1.2 eV), high electrical conductivity, and strong catalytic activity, enabling applications in energy storage, sensing, and environmental remediation [1,3,5,6]. Despite these advantages, uncontrolled aggregation, oxidation, and morphology-dependent behavior continue to constrain their performance. Therefore, precise control over nanoparticle size, aggregation state, phase composition, and oxidation is essential for tailoring Cu_2_O and CuO nanomaterials to specific functional applications. Accordingly, oxide formation is not necessarily undesirable; the preferred Cu/Cu_2_O/CuO phase balance depends on the intended application, with metallic Cu favored where high electrical conductivity is required and Cu_2_O/CuO phases potentially beneficial where semiconducting, catalytic, or surface-active properties are desired.

To achieve such control, synthesis strategies have focused on reproducibility and phase selectivity. Conventional wet-chemical approaches, including coprecipitation, sol–gel, hydrothermal, and microwave-assisted routes, are widely employed due to their versatility and relative simplicity [7]. These approaches enable the modulation of crystal structure and oxidation state through the adjustment of reaction parameters such as pH, temperature, precursor concentration, and atmosphere. However, they commonly require surfactants or stabilizing ligands to regulate nucleation and growth, which may introduce surface contamination or residual species that influence interfacial chemistry and limit applications requiring clean, highly reactive surfaces. Moreover, achieving reproducible phase selectivity between Cu, Cu_2_O, and CuO remains challenging, particularly under scale-up conditions [8]. Physical routes such as laser ablation, evaporation–condensation, and vapor deposition yield high-purity nanoparticles without chemical contamination. Despite these advantages, their reliance on high energy input and complex instrumentation often constrains throughput and industrial scalability [9,10,11]. In response to increasing environmental and economic concerns, green synthesis strategies have recently gained significant attention. These methods utilize biological reducing and stabilizing agents derived from plants or microorganisms, thereby reducing hazardous chemical usage. While environmentally attractive, such strategies frequently offer limited control over size distribution, crystallinity, and phase purity, and may introduce organic residues that affect surface-dependent performance [12].

Spark ablation represents a complementary dry, gas-phase approach that addresses many of these limitations. In this process, repetitive high-voltage discharges between two electrodes create transient plasma conditions that vaporize small quantities of electrode material. The resulting metal vapor cools rapidly, forming clusters and nanoparticles that are transported downstream as an aerosol and can be deposited directly onto substrates [13,14,15]. Because the process requires only solid electrodes, electrical power, and carrier gas, it provides a relatively simple, contamination-free, and continuous route to nanoparticle production. Moreover, operational parameters such as discharge energy, carrier-gas composition, deposition time, and flow rate can influence particle size, aggregation, and oxidation behavior, while the resulting Cu/Cu_2_O/CuO phase composition also depends strongly on the oxidation environment. This combination of cleanliness, scalability, and process control makes spark ablation particularly attractive for producing copper-based nanoparticles tailored for functional applications [9].

Nanoparticle formation during spark ablation proceeds through nucleation, growth, collision, and aggregation within the carrier-gas stream. The carrier-gas flow rate governs the residence time of nanoparticles and clusters within the aggregation zone and therefore influences the extent to which collision, coalescence, and structural relaxation can proceed before deposition. At lower flow rates, the longer residence time provides greater opportunity for colliding particles and clusters to undergo coalescence and restructuring, favoring more compact, near-isotropic agglomerates. As the flow rate increases, the shorter residence time limits the extent of such restructuring before deposition, so that particle and cluster attachment becomes increasingly kinetically constrained and more open, elongated, chain-like structures can be retained [10,11,13,14,16,17,18]. The observed morphology therefore reflects the competition between the timescale available for gas-phase transport and the timescales associated with coalescence and structural relaxation. For reactive metals such as copper, these aggregation dynamics are closely coupled with oxidation processes, since particle size and agglomerate structure affect surface area available for post-deposition oxide formation [19,20]. Throughout this work, the term aggregation refers to the collision and attachment of nanoparticles into larger agglomerates, whereas coalescence denotes the subsequent structural relaxation and partial fusion of contacting nanoparticles, resulting in more compact agglomerate morphologies.

In our previous work on spark-ablated bismuth telluride (Bi–Te) nanoparticles, we systematically investigated the influence of the electrical discharge parameters and demonstrated that both voltage and current affect primary nanoparticle size, with lower values producing smaller nanoparticles under the investigated conditions. These results highlight the role of the discharge conditions in determining the initial particle population generated by spark ablation. An optimal carrier-gas flow rate was also found to yield a more homogeneous agglomerate distribution [21]. Although these findings demonstrate that spark-ablation parameters can influence particle size and agglomeration behavior, copper behaves differently from Bi–Te due to its higher reactivity and propensity for oxidation, necessitating a dedicated investigation into its gas-phase synthesis dynamics.

Carrier-gas composition represents an additional process parameter, as the physical and chemical properties of the gas can influence both the discharge characteristics and the subsequent formation and evolution of the nanoparticles. Changes in carrier-gas composition have been reported to modify the breakdown behavior, particle size distribution and, particularly in reactive systems, nanoparticle composition and oxidation state [22,23]. In the present study, high-purity N_2_ was therefore maintained as a fixed carrier gas to isolate the influence of flow rate, while systematic comparison with other carrier gases remains an important direction for future investigation.

In this work, copper nanoparticles were generated by spark ablation and deposited onto silicon and glass substrates under the same controlled nitrogen flow conditions. Silicon substrates were used primarily for scanning electron microscopy (SEM) characterization of the deposited morphology, while material recovered from the Si substrates was subsequently transferred onto carbon-supported grids for transmission electron microscopy (TEM), fast Fourier transform (FFT), and selected area electron diffraction (SAED) analyses. Glass substrates were used for Raman spectroscopy to enable spectroscopic characterization of the deposited copper oxide material.

Carrier-gas flow rate and deposition time were systematically varied while maintaining constant spark power to isolate the influence of gas-phase transport and deposition duration on particle size, agglomeration, and deposition morphology. Within this experimental framework, carrier-gas flow rate primarily modifies the transport and residence conditions governing agglomerate restructuring, whereas deposition time primarily determines the extent of nanoparticle accumulation on the substrate. Copper nanoparticles exhibit high surface reactivity, and even under oxygen-limited conditions, oxidation can occur rapidly upon ambient exposure [24,25,26]. In our experiments, deposition was conducted using a diffusion-based deposition configuration that promotes particle transport and deposition under high-purity N_2_. Oxidation may occur both during aerosol transport and following ambient exposure, since even trace levels of residual oxygen can induce oxidation of highly reactive nanoparticles. The present measurements do not allow for the relative contributions of in-flight and post-deposition oxidation to be quantitatively separated. The objective of this study is to investigate how carrier-gas flow rate influences nanoparticle agglomeration under the investigated deposition conditions and to assess the oxide phase characteristics following ambient exposure.

## 2. Experimental Methods

### 2.1. Nanoparticle Deposition and Sample Preparation

**Figure 1:**
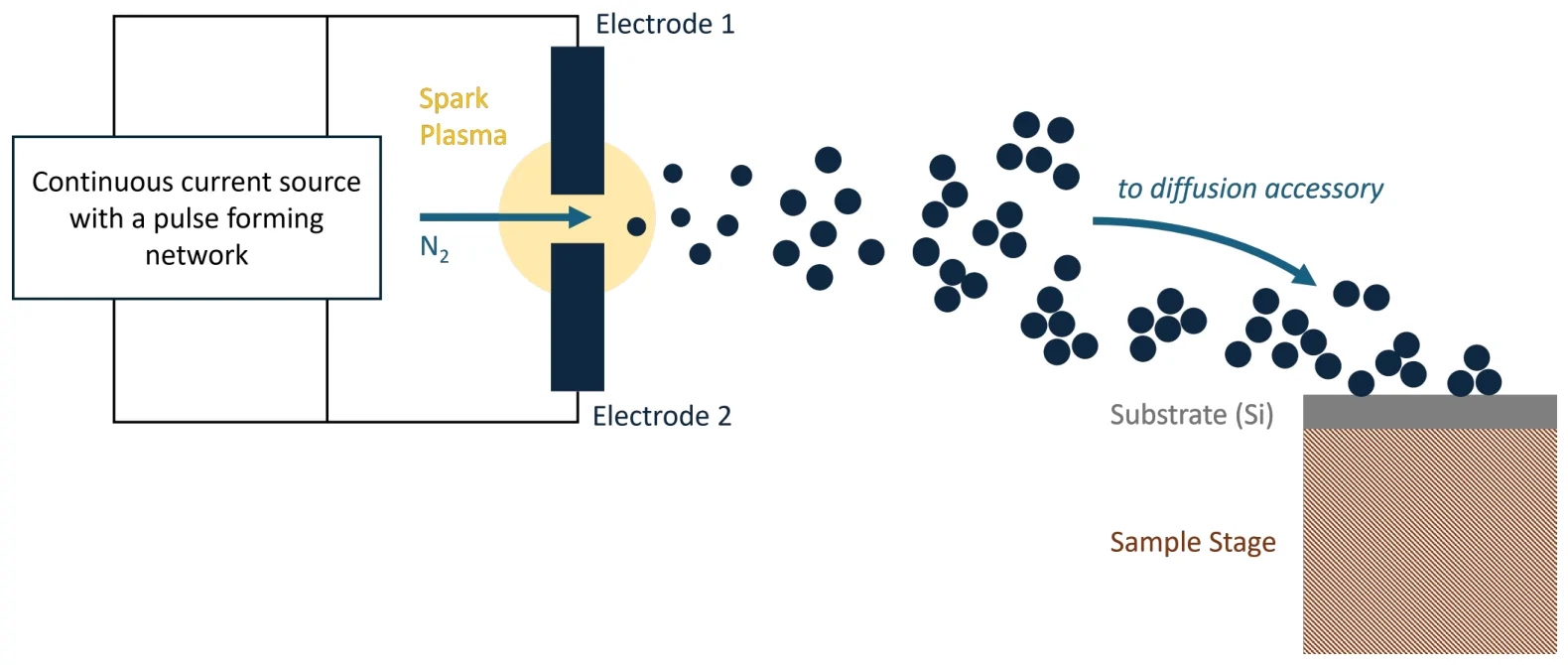
Schematic illustration of the spark ablation nanoparticle generation and deposition system. The different circle sizes schematically represent particles and agglomerates of varying sizes; the drawing is not to scale.

Cu nanoparticles were generated using a VSPARTICLE spark ablation generator (VSP-G1; VSParticle B.V., Delft, The Netherlands) equipped with a diffusion accessory and deposited onto silicon (Si) and glass substrates (surface dimensions of 1 cm × 1 cm) under a high-purity N_2_ (99.999%) carrier gas (Figure 1). The diffusion-based deposition configuration produces spatially distributed nanoparticle agglomerates on the substrate rather than a continuous, uniform film; therefore, a single representative film thickness is not defined for these samples. The spark generator was operated at a constant spark power of 9.1 W for all sample series variations, corresponding to a voltage of 1.3 kV and a current of 7 mA. Although higher currents were used in our previous study on bismuth telluride [21], in our experiments, copper electrodes exhibited unstable sparking at elevated currents, which we attribute to their high thermal and electrical conductivity. Consequently, a current of 7 mA was selected to ensure consistent deposition across all samples.

After deposition, samples were exposed to ambient conditions prior to characterization. Oxidation may occur during aerosol transport because trace residual oxygen can react with the high-surface-area Cu nanoparticles, and further oxidation may occur following exposure to ambient air after deposition. The present experimental design does not allow for the relative contributions of these oxidation stages to be quantitatively separated.

Similar to our previous work with Bi_2_Te_3_ nanoparticles [21], to examine the influence of carrier gas flow on nanoparticle transport and surface accumulation, a first series of samples was prepared with a fixed deposition time of 2 h while varying the N_2_ flow rate between 0.5 L min^−1^ and 3.5 L min^−1^. Based on initial observations of surface morphologies via scanning electron microscopy (SEM), which identified 0.5 L min^−1^ and 1.5 L min^−1^ as yielding the most uniform and representative agglomerate structures after 2 h, these two flow conditions were selected for a second series in which the deposition time was extended to 5 h to probe the effect of prolonged nanoparticle accumulation; higher flow rates (2.5 L min^−1^ and 3.5 L min^−1^) were not extended to 5 h to avoid excessive overlap and non-uniform surface coverage observed in the preliminary 2 h series. No post-deposition treatments were applied. The full set of sample series variations and deposition parameters is summarized in Table 1.

### 2.2. Characterization Techniques

Surface morphology was examined using a JEOL Neoscope JCM-7000 (JEOL Ltd., Akishima, Japan) scanning electron microscope (SEM) in high-vacuum mode using a secondary electron detector (SED) at an accelerating voltage of 5.0 kV. Overview images were acquired at low magnification to evaluate coverage and agglomerate distribution across the substrate. Higher-magnification images were obtained to examine individual agglomerate morphology and agglomerate-scale surface texture. Individual nanoparticles were not resolved at these magnifications due to their small size and low contrast on the Si substrate.

Agglomerates were identified as contiguous clusters of deposited material appearing as single connected objects. A total of 296 agglomerates were measured across multiple randomly selected regions. For each agglomerate, the maximum projected length and the corresponding perpendicular width were measured using the instrument’s native software. This two-parameter approach captures both agglomerate size and shape, allowing compact, roughly isotropic agglomerates to be distinguished from elongated, chain-like structures without reducing their geometry to a single scalar metric. Identical measurement criteria were applied across all samples. Additional large-area and higher-magnification SEM micrographs from each carrier-gas flow-rate condition are provided in Figure S9 to document the surface distribution and recurrence of the observed morphologies.

Elemental analysis was performed using the integrated silicon drift detector (SDD) energy-dispersive X-ray spectroscopy (EDS) system incorporated within the SEM. Point spectra and elemental maps for Cu were collected from representative regions of each sample under high-vacuum conditions at an accelerating voltage of 15 kV. Oxygen signals were interpreted with caution due to the presence of a native SiO_2_ layer on the Si substrates, which contributes to the O Kα emission. No conclusions regarding the Cu oxidation state or stoichiometry were drawn from the EDS data; these aspects were instead assessed using Raman spectroscopy, FFT analysis, and SAED.

Structural characterization of the constituent nanoparticles was carried out using a JEOL ARM200CF (JEOL Ltd., Akishima, Japan) scanning transmission electron microscope (STEM) equipped with spherical aberration correction on both the probe and objective lenses and operated at an acceleration voltage of 200 kV. Samples were prepared by mechanically scraping the deposited nanoparticles from the substrate with a fine diamond tip and transferring them onto carbon membranes supported by a Cu grid. Because this mechanical transfer procedure can disrupt weakly bound, micrometer-scale deposited agglomerates, the TEM analysis was used primarily to characterize the constituent primary nanoparticles and their local nanoscale organization rather than to reproduce the intact agglomerate morphology observed directly on the substrate by SEM. High-resolution TEM imaging enabled direct visualization of individual nanoparticles and analysis of their size distribution, morphology, and crystallinity. FFT and SAED analyses were performed on selected regions to identify lattice spacings and crystalline phases.

Raman spectra were acquired using a Renishaw InVia Reflex spectrometer (Renishaw plc, Wotton-under-Edge, UK) coupled to a Leica optical microscope (Leica Microsystems GmbH, Wetzlar, Germany) in a micro-Raman configuration. Excitation was provided by a He–Ne laser (λ = 632.8 nm) with an 1800 mm^−1^ grating in a backscattering geometry. A 50× objective lens was used, and all measurements were performed at ambient temperature. Laser power was adjusted to maximize signal intensity while avoiding local heating or sample modification. Measurements were performed at different laser powers and at multiple locations on the deposited material to assess signal stability and spatial reproducibility. Spectra acquired at the investigated laser powers showed the same characteristic CuO- and Cu_2_O-related bands without additional features or systematic spectral changes, indicating no detectable laser-induced phase transformation within the investigated power range. Each spectrum was collected with an integration time of 180 s per accumulation and three accumulations per measurement. Wavenumber calibration was performed using the Si reference peak at 520.5 cm^−1^. Reference Raman spectra of the bare glass substrate and CuO powder were also acquired under comparable measurement conditions for comparison with the deposited sample.

## 3. Results and Discussion

**Figure 2:**
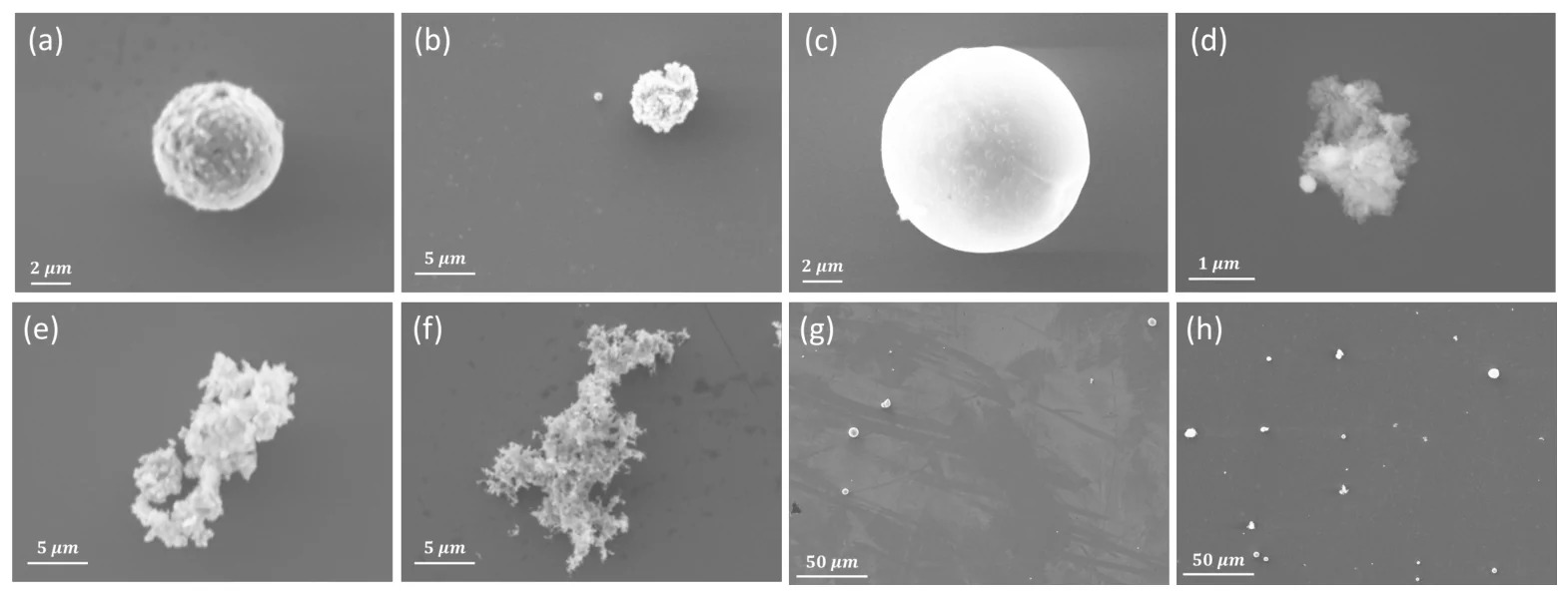
Variations in agglomerate morphology observed for 2 h deposition as a function of N_2_ carrier gas flow rate: (**a**,**b**) 0.5 L min^−1^, (**c**,**d**) 1.5 L min^−1^, (**e**) 2.5 L min^−1^, and (**f**) 3.5 L min^−1^. Comparison at 1.5 L min^−1^ for deposition times of (**g**) 2 h and (**h**) 5 h shows increased surface coverage with minimal change in agglomerate morphology.

To study agglomerate-level morphology, the deposited Cu material was imaged by SEM on silicon substrates, chosen for their conductivity and stable contrast. As seen in Figure 2a–f, the deposited Cu agglomerates exhibit two recurring morphologies across all selected carrier gas flow rates and deposition times: compact, near-isotropic clusters and elongated, chain-like assemblies. The compact agglomerates appear as dense clusters with comparable projected length and width (Figure 2a,c), whereas the elongated structures display pronounced anisotropy, with lengths significantly exceeding their widths (Figure 2b,d–f). The latter form open, branched networks composed of loosely connected sub-clusters, giving rise to filamentary or cloud-like morphologies. For 2 h depositions, both morphologies are observed across the investigated conditions.

As the N_2_ carrier gas flow rate is increased to 3.5 L min^−1^, a clear shift in agglomerate morphology is observed. At lower flow rates (0.5 L min^−1^ to 1.5 L min^−1^), the deposited material is dominated by compact agglomerates (Figure 2a–d), whereas higher flow rates (2.5 L min^−1^ to 3.5 L min^−1^) increasingly favor elongated, chain-like structures with larger length-to-width ratios (Figure 2e–f). In spark ablation and gas aggregation systems, the carrier gas flow rate controls the residence time of nanoparticles and clusters within the aggregation zone prior to deposition [21]. Lower effective flow rates allow particles to remain longer in the aggregation region, increasing the probability of collision, coalescence, and partial structural relaxation before deposition, whereas higher flow rates shorten the available residence time and limit the extent of coalescence and structural relaxation before deposition, favoring increasingly kinetically constrained aggregation pathways [27,28]. High flow rates were excluded from further experiments because elongated, filamentary agglomerates dominated, reducing the reproducibility of compact clusters, as confirmed by their projected dimensions and aspect ratios. In the low-flow regime, agglomerates at 1.5 L min^−1^ were the most uniform and closely resembled those formed at 0.5 L min^−1^, providing the highest morphological consistency across the explored parameter space.

The irregular and elongated structures observed by SEM are consistent with agglomerates assembled from nanoscale primary particles; however, SEM morphology alone cannot unambiguously distinguish the most compact, nearly spherical features from occasional resolidified droplets generated by spark splashing. We therefore cannot exclude a splash-particle contribution to some of the larger rounded features. TEM characterization of material recovered from the substrate nevertheless shows that the deposited material contains predominantly nanoscale, rounded primary particles, supporting the interpretation that the irregular and chain-like SEM features arise from assemblies of these primary nanoparticles.

Additional SEM micrographs acquired from multiple substrate regions are presented in Figure S9. They show recurring compact, irregular, and elongated micrometer-scale deposited structures, together with smaller isolated features. These observations characterize the morphology after aerosol transport and diffusion-based deposition and should not be interpreted as a direct measurement of the gas-phase aerosol output of the spark generator. At the available SEM resolution, the individual, approximately 10 nm primary particles within these deposits cannot be resolved; their size is determined independently by TEM.

Extending the deposition time to 5 h at 0.5 L min^−1^ and 1.5 L min^−1^ increased surface coverage while preserving the dominant agglomerate morphologies (Figure 2g,h). Comparison with our previous study on spark-ablated Bi–Te nanoparticles under comparable carrier-gas flow conditions indicates material-dependent differences in the resulting deposited morphology [21]. For Cu, limited post-deposition surface diffusion and coalescence may additionally contribute to the observed agglomerate morphology, consistent with previous studies of metallic nanoparticle surface diffusion and coalescence [28,29,30,31,32].

**Figure 3:**
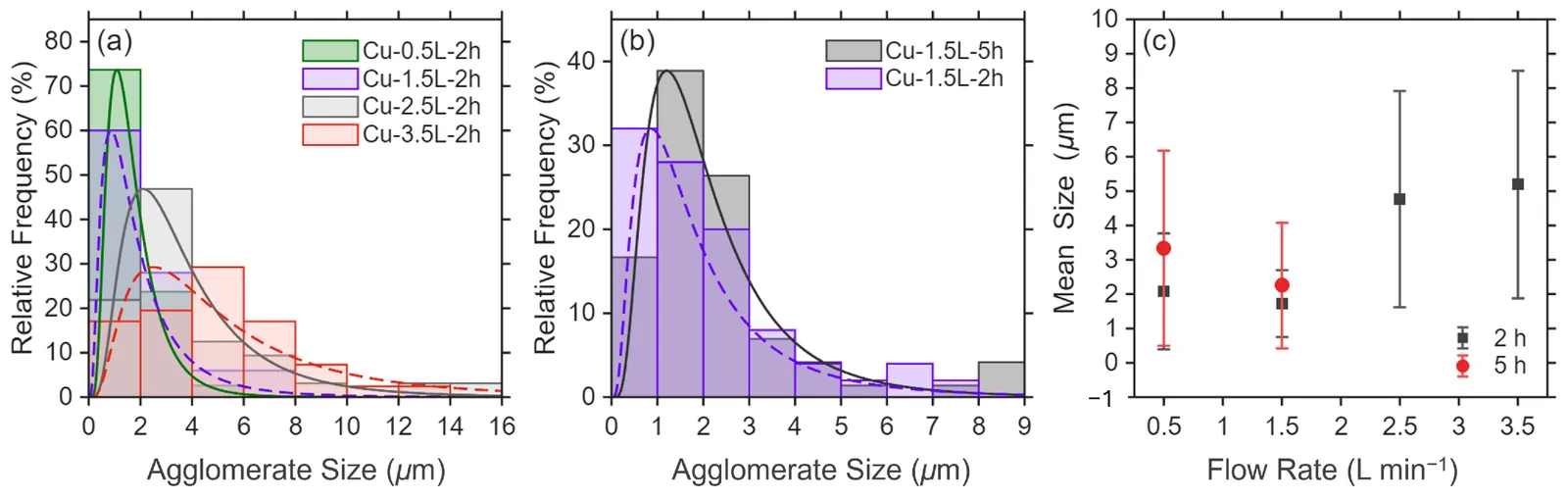
Quantitative analysis of Cu agglomerate size as a function of N_2_ carrier gas flow rate and deposition time. Solid and dashed curves show lognormal fits to the corresponding datasets, scaled to the maximum height of each histogram; the line styles distinguish overlapping curves. (**a**) Agglomerate size distributions for flow rates of 0.5 L min^−1^, 1.5 L min^−1^, 2.5 L min^−1^ and 3.5 L min^−1^, showing a transition from narrow, compact distributions at low flow to broader distributions at higher flow rates. (**b**) Comparison of agglomerate size distributions at 1.5 L min^−1^ for deposition times of 2 h and 5 h. (**c**) Agglomerate mean size at 0.5 L min^−1^, 1.5 L min^−1^, 2.5 L min^−1^ and 3.5 L min^−1^ for 2 h deposition and at 0.5 L min^−1^ and 1.5 L min^−1^ for 5 h deposition. Error bars represent one standard deviation of the measured agglomerate dimensions calculated from the corresponding population of individual agglomerates for each deposition condition.

The previously described flow- and time-dependent trends in agglomerate morphology are quantitatively supported by measurements of projected dimensions across all samples, as summarized in Figure 3. Figure 3a shows the agglomerate size distributions of the Cu samples synthesized for 2 h at different flow rate values. The Cu-0.5L-2h and Cu-1.5L-2h samples exhibit narrow, unimodal distributions centered at approximately 1 µm, with most agglomerates confined below 3 µm and only a minor fraction extending toward larger sizes, indicating limited secondary aggregation and a relatively homogeneous population.

In contrast, increasing the flow rate to 2.5 and 3.5 L min^−1^ produces broader distributions shifted toward larger agglomerate dimensions, with pronounced right-skewed tails extending well beyond the dominant low-size population.

At 1.5 L min^−1^, extending the deposition time from 2 h to 5 h broadens the distribution and increases the contribution of larger agglomerates, while the dominant modal region remains at approximately the same agglomerate size. The corresponding mean size increases from approximately 1.7 to 2.3 µm. This behavior is consistent with previous reports describing time-dependent agglomerate growth and restructuring during nanoparticle deposition [33].

Taken together, Figure 3c quantitatively summarizes this behavior by presenting the mean agglomerate size as a function of flow rate for 2 h and 5 h depositions. At 2 h, the mean projected agglomerate size remains near 2 µm at the two lower flow rates (0.5 and 1.5 L min^−1^), before increasing to approximately 4.8 and 5.2 µm at 2.5 and 3.5 L min^−1^, respectively. The increasing standard deviation at elevated flow rates further indicates greater size dispersion and structural heterogeneity. For the two low-flow conditions examined at 5 h, the mean agglomerate size is higher than the corresponding value at 2 h, consistent with the broader distributions observed after extended deposition.

A similar flow-associated transition from compact to more extended agglomerates was observed in our previous study of spark-ablated Bi–Te nanoparticles [21], indicating that carrier-gas transport conditions can influence agglomerate morphology across different material systems, while the detailed response remains material-dependent.

At the lowest flow rates investigated, the predominance of compact, approximately isotropic agglomerates is therefore consistent with extended gas-phase residence times that enable enhanced collision frequency and partial structural relaxation prior to deposition. In addition, physically synthesized Cu nanoparticles exhibit appreciable surface mobility upon substrate impact, allowing for limited post-deposition restructuring that can further smooth agglomerate outlines [34]. As the flow rate increases to intermediate values, agglomerates retain an overall compact morphology but display increased surface roughness and shape irregularity, consistent with progressively reduced opportunity for complete coalescence and restructuring before deposition [27]. At the highest flow rates (2.5 L min^−1^ to 3.5 L min^−1^), the emergence of elongated, chain-like agglomerates indicates aggregation under increasingly kinetically constrained conditions, producing open, elongated structures with filamentary morphologies [35]. Overall, the evolution of agglomerate morphology and size reflects the competition between the available gas-phase residence time and the extent of coalescence and structural relaxation that can occur before deposition, with additional surface-mediated restructuring possible after substrate impact [27,28,34,35].

For Cu specifically, the high surface mobility of metallic nanoparticles upon substrate impact facilitates limited diffusion-driven coalescence and local densification [34], moderating structural heterogeneity without inducing a morphological regime change. For further analysis, we select the 1.5 L min^−1^ condition as it offers optimal surface coverage and particle uniformity.

**Figure 4:**
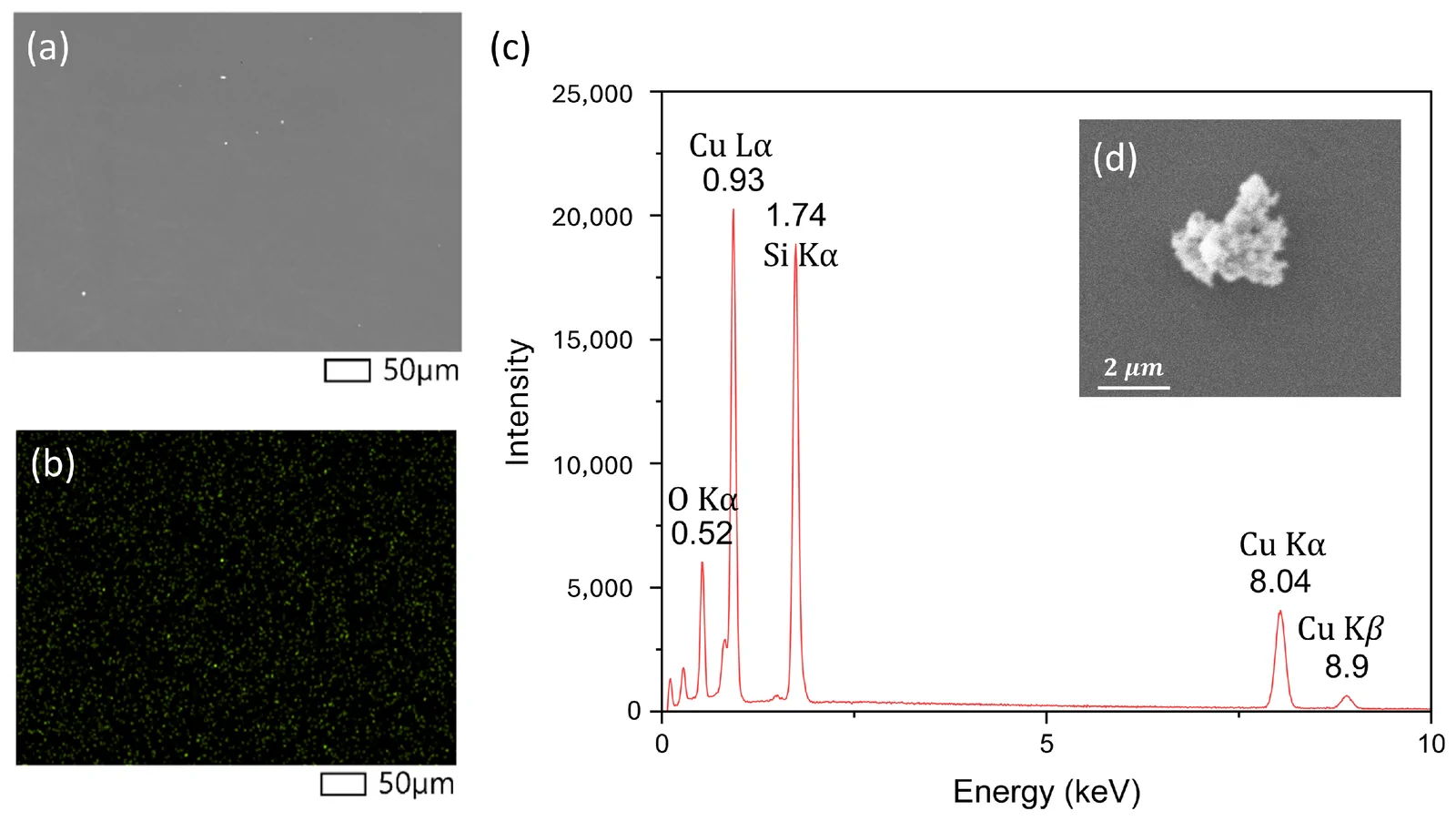
Elemental analysis of Cu agglomerates on a Si substrate. (**a**) SEM image of a representative surface region indicating the area selected for EDS analysis. (**b**) Corresponding EDS elemental maps of Cu, showing the spatial distribution across the agglomerates and surrounding substrate. (**c**) EDS point spectra acquired from the agglomerate highlighted in (**d**), confirming its Cu-rich composition.

The elemental composition of the deposited features was examined by SEM coupled with EDS, as illustrated in Figure 4. EDS mapping shows that Cu is spatially associated with the observed agglomerates, while point spectra confirm their Cu-rich composition. Oxygen is also detected; however, its signal cannot be unambiguously attributed to Cu oxidation because of the contribution from the native SiO_2_ layer on the substrate. The spectra exhibit the expected O Kα, Cu Lα, Si Kα, and Cu Kα/Kβ peaks [36,37]. Accordingly, EDS is used here as complementary elemental characterization rather than for determination of Cu oxidation state or phase composition.

**Figure 5:**
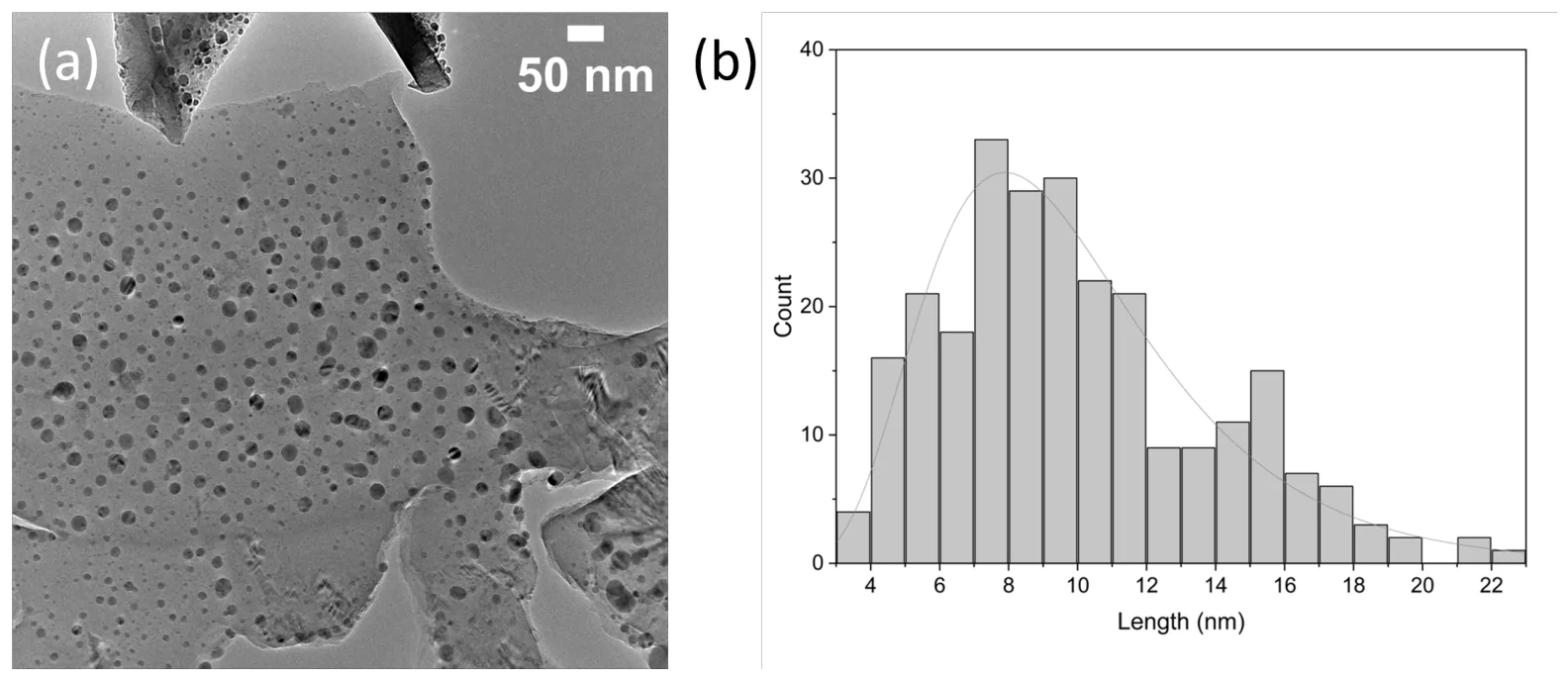
(**a**) TEM image showing the shape and morphology of the constituent primary Cu nanoparticles. (**b**) Histogram illustrating the nanoparticle diameter distribution, showing a predominantly small-diameter population.

To distinguish the constituent primary nanoparticles from the micrometer-scale structures observed by SEM, TEM was used to resolve and quantify the size and morphology of the individual nanoparticles, as illustrated in Figure 5. Images were acquired at multiple randomly selected regions, and particle diameters were measured manually using calibrated ImageJ software (version 1.54g), yielding a total of 260 particles for statistical analysis. The SEM and TEM populations therefore represent independent measurements at different structural levels: SEM quantifies deposited agglomerates, whereas TEM quantifies the constituent primary nanoparticles. The nanoparticles appeared circular or near-circular in shape (Figure 5a), confirming the predominantly spherical morphology observed across the sample.

The size distribution, presented as a histogram in Figure 5b, shows a mean size of 10 nm ± 4 nm and a right-skewed profile, characterized by a dominant population centered at the smaller diameters and a trailing tail extending toward larger diameters (>15 nm). This asymmetry indicates that most particles formed via primary nucleation and growth, while the larger tail reflects particles generated through stochastic collisions, coalescence, or aggregation events during gas-phase transport and deposition, as commonly observed in spark ablation-generated aerosols [13,27,38,39,40,41]. The nanometer-scale dimensions measured by TEM therefore refer to the constituent primary particles and should not be directly compared with the micrometer-scale projected dimensions of the deposited agglomerates measured by SEM. Because the TEM specimens were prepared by mechanically removing the deposited material from the substrate, intact micrometer-scale agglomerate architecture is not necessarily preserved during transfer.

Copper’s relatively high surface energy may additionally favor coalescence and restructuring of the nanoscale particles during transport and deposition, contributing to the observed size distribution.

**Figure 6:**
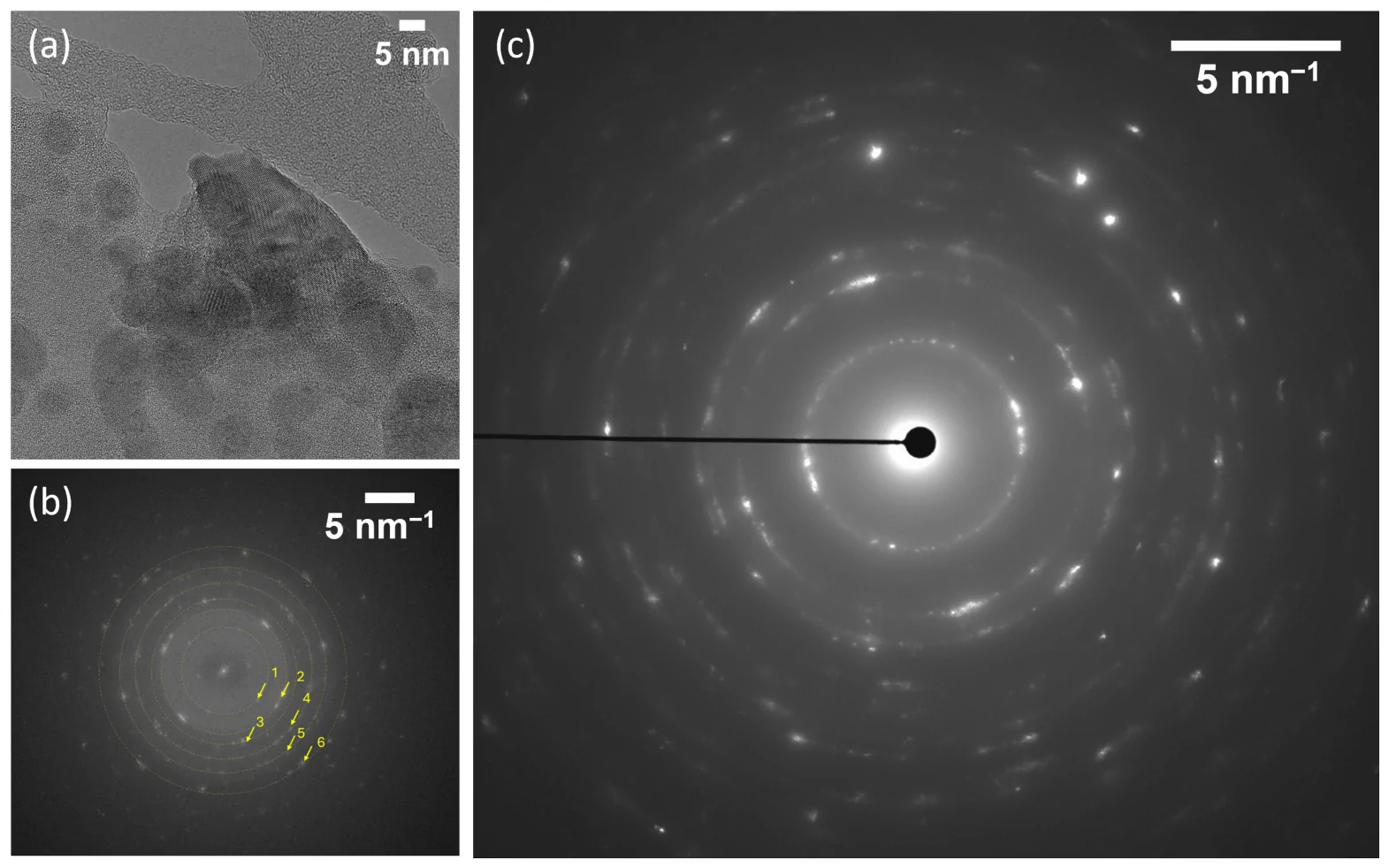
(**a**) High-resolution TEM (HRTEM) image of the copper oxide nanoparticle ensemble, showing agglomerated nanostructures. (**b**) Fast Fourier transform (FFT) of the selected HRTEM region, with highlighted rings and sampling points used for lattice spacing analysis. (**c**) Selected area electron diffraction (SAED) pattern acquired from a representative nanoparticle ensemble, showing concentric diffraction rings characteristic of a nanocrystalline layer on a Si substrate, with the diffraction signal dominated by substrate contributions within the selected area aperture. The FFT analysis probes local crystalline domains, while the SAED pattern reflects the ensemble-averaged diffraction response dominated by the substrate contribution.

Local crystallinity and lattice structure were investigated using high-resolution TEM images, which were subjected to FFT analysis to extract spatial frequency information associated with lattice periodicities (Figure 6a,b). FFT patterns generated from selected regions exhibited distinct diffraction spots indicative of crystalline order. Spatial frequencies were measured by quantifying the radial distance between the central peak and surrounding maxima in the FFT image. These values, expressed in reciprocal nanometers, correspond to the inverse of real-space lattice spacings.

The corresponding lattice spacings were calculated using d=1/f, where *f* is the spatial frequency and *d* is the lattice spacing in nanometers. The extracted lattice spacings can support phase assignment through comparison with reference values for metallic Cu and Cu_*x*_O phases. Deviations from pure metallic Cu spacings may indicate partial oxidation or mixed-phase formation, common in high-surface-area nanostructures exposed to ambient conditions.

FFT analysis of the HRTEM images (Table 2) reveals lattice spacings consistent with monoclinic CuO within the locally sampled regions [42]. In particular, the observed lattice spacing at 2.27 ± 0.06 Å is most consistent with the principal diffraction peak of CuO. The remaining observed spacings exhibit lower prominence and are therefore considered minor contributions, although they are systematically identified and assigned where possible. While some spacings may overlap with Cu or Cu_2_O reflections, no prominent Cu_2_O spacings, such as those near 2.46, 2.13, 1.74, or 1.51 Å [42], are clearly resolved in the analyzed regions. Additional FFT analyses from multiple regions are provided in the Supplementary Information (Figures S4–S6 and Table S2), confirming the reproducibility of the extracted lattice spacings. Although minor contributions from other phases cannot be excluded below the detection limit, particularly in regions of overlapping d-spacings, the FFT results indicate that the nanoscale structure resolved in this field of view is predominantly consistent with CuO.

To assess the broader crystallographic environment beyond the local FFT analysis, selected area electron diffraction (SAED) patterns were acquired from representative nanoparticle ensembles over a larger sampled area across multiple regions (Figure 6c). Radial intensity profiles were processed using ImageJ, yielding averaged d-spacings in the ranges summarized in Table 3, including prominent values near 3.1–3.2 Å, 2.03–2.14 Å, 1.92–1.97 Å, and 1.63–1.66 Å. These values were compared with reference diffraction data for Si and Cu-containing phases, as summarized in Table 3. Additional SAED patterns acquired from multiple regions are provided in the Supplementary Information (Figures S1–S3), confirming the reproducibility of the substrate-dominated diffraction response.

Comparison with reference crystallographic data [42] (Table 3) shows that the majority of the measured spacings correspond to Si reflections, including those near 3.14 Å (111), 1.92 Å (220), 1.64 Å (311), 1.36 Å (400), and 1.25 Å (331), confirming a dominant contribution from the substrate. Features in the 2.03–2.14 Å range do not correspond to Si and are consistent with Cu-containing phases, including metallic Cu (111) and Cu_2_O; however, these cannot be uniquely assigned due to peak overlap. A weak feature near ∼2.54 Å is also observed, but its low intensity and lack of supporting reflections prevent reliable phase identification. Importantly, for certain d-spacings, both discrete spots and diffuse ring-like features are observed at the same scattering vectors (Figure 6c). This indicates the coexistence of contributions from well-oriented crystalline domains (attributed primarily to the Si substrate) and nanocrystalline or randomly oriented regions within the probed area.

Overall, the SAED patterns do not correspond to a single, well-defined phase and are strongly dominated by diffraction from the Si substrate, with only limited and non-unique contributions from Cu-containing phases. These limitations arise from both peak overlap and the contribution of the Si support within the selected area aperture, preventing unambiguous phase identification from SAED alone.

This highlights the complementary nature of local (FFT) and ensemble (SAED) analyses: the FFT results indicate that locally resolved regions are consistent with CuO, whereas the ensemble diffraction response is dominated by the substrate and does not allow for definitive distinction between CuO and Cu_2_O. Accordingly, SAED does not contradict the CuO assignment but lacks the phase selectivity required for conclusive identification.

Raman spectroscopy was therefore employed to complement the local (FFT) and ensemble (SAED) structural analyses as a phase-selective vibrational probe of copper oxide phases, distinguishing metallic copper from its oxides. Its selectivity allows for substrate contributions to be minimized under appropriate conditions, enabling more definitive identification of oxide phases.

**Figure 7:**
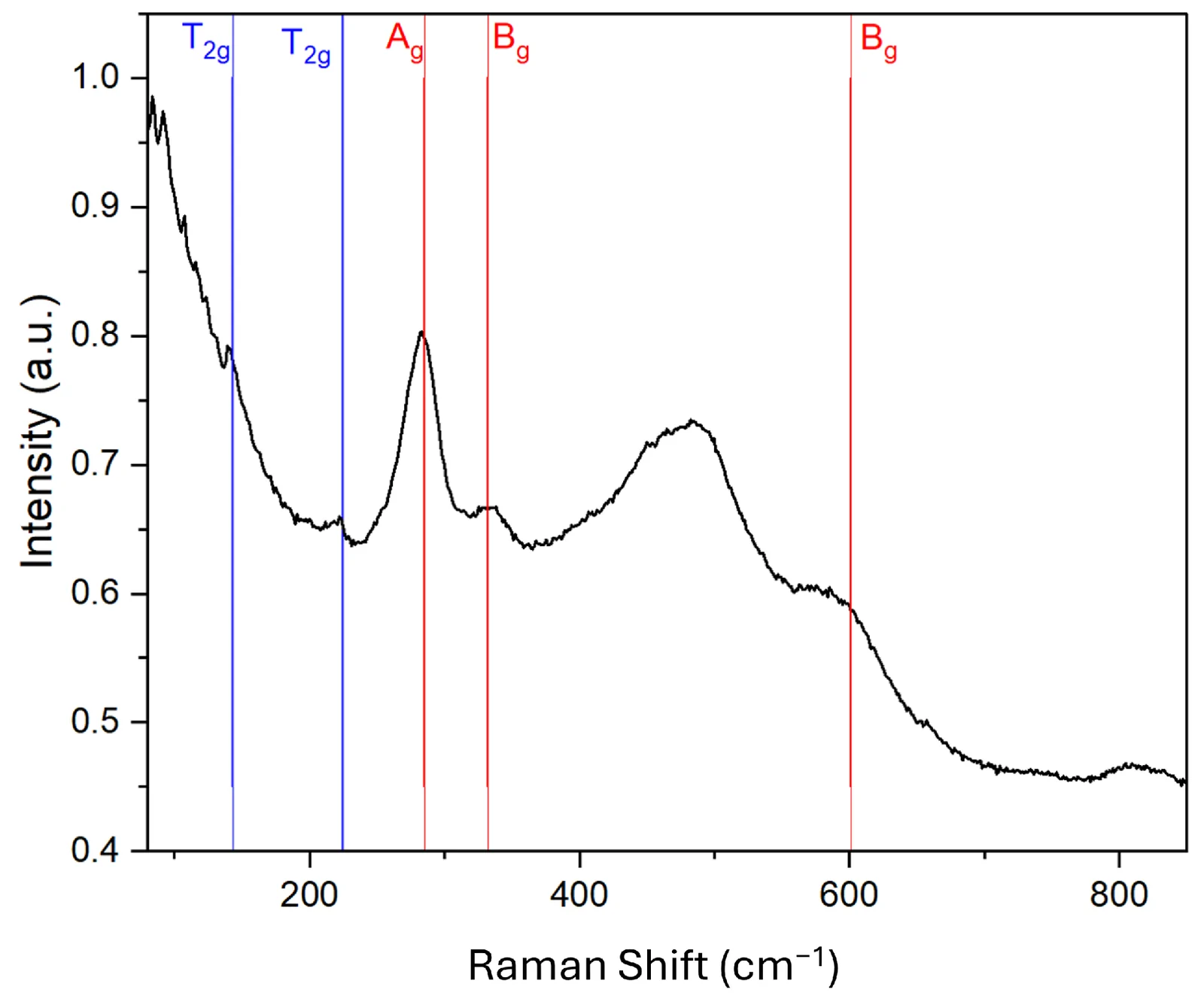
Raman spectra of nanostructured copper oxide particles. Characteristic vibrational modes of CuO are labeled in red, while those of Cu_2_O are labeled in blue.

Figure 7 presents the Raman spectra of the nanostructured copper oxide layers. Distinct Raman bands corresponding primarily to CuO, with minor contributions from Cu_2_O, are observed, along with features originating from the underlying amorphous glass substrate.

Reference measurements of the bare glass substrate and CuO powder were performed to support the assignment of the observed Raman features. The bare glass spectrum confirms the broad substrate-related contribution, whereas comparison with the CuO powder reference supports the assignment of the principal CuO-related bands in the deposited material. Reference Raman spectra of the bare glass substrate, together with comparison spectra of the deposited sample, glass substrate and CuO powder, are provided in the Supplementary Information (Figures S7 and S8, respectively).

However, the present measurements do not establish a systematic dependence of the Cu_2_O/CuO phase balance on carrier-gas flow rate or deposition time. Because oxidation may occur both during aerosol transport and following ambient exposure, the present measurements do not allow the relative contributions of in-flight and post-deposition oxidation to the observed CuO/Cu_2_O phase characteristics to be quantitatively separated.

Because metallic Cu is Raman-inactive, the Cu-related Raman bands are attributed to oxide phases. Characteristic CuO modes appear at approximately 285, 332, and 601 cm^−1^, while weaker Cu_2_O-related features are observed near 143 and 225 cm^−1^. Comparable Raman signatures have been reported for copper oxide nanoparticles synthesized by flame spray pyrolysis, with similar peak positions for both CuO and Cu_2_O phases [28,43]. CuO crystallizes in a monoclinic structure with space group C2/c, in which each Cu atom is coordinated by four oxygen atoms forming a distorted rectangular geometry [44]. Group-theoretical analysis yields the zone-center optical phonon representation

$$\Gamma =4A_{u}+5B_{u}+A_{g}+2B_{g}$$

where the $A_{g}$ and $2B_{g}$ modes are Raman-active, while the remaining modes are infrared active or acoustic and therefore do not contribute to the Raman response [26,45]. Accordingly, the Raman band observed at ∼285 cm^−1^ is assigned to the $A_{g}$ mode, and the bands at ∼332 and ∼601 cm^−1^ correspond to the $B_{g}$ phonon modes of monoclinic CuO. These assignments are consistent with Raman spectra reported for bulk and nanocrystalline CuO, where the characteristic modes are typically observed at ∼288–296 cm^−1^ ($A_{g}$), ∼330–344 cm^−1^ ($B_{g}-1$), and ∼610–635 cm^−1^ ($B_{g}-2$) [43,46,47].

Consistent with these vibrational assignments, the CuO-related Raman bands in the present nanostructured layers exhibit pronounced broadening and a systematic red shift toward lower wavenumbers relative to bulk CuO. Such red shifts are commonly associated with reduced crystallite size, enhanced structural disorder, and phonon confinement within nanoscale domains, which relax the momentum conservation rules governing first-order Raman scattering [48,49,50,51,52]. Additional contributions from defect-induced phonon localization may influence the observed frequency shifts and linewidth broadening, in agreement with previous reports documenting enhanced spin–phonon coupling with decreasing nanostructure dimensions [49,53]. Alongside the CuO-related bands, low-intensity features at ∼143 and ∼225 cm^−1^ are attributed to the Cu_2_O phase, in agreement with earlier reports [54,55], reflecting its minor presence. Cu_2_O crystallizes in a cubic cuprite structure with space group Pn$\bar{3}$m with six atoms per unit cell, resulting in 18 zone-center phonon modes described by [56]:

$$\Gamma =A_{2u}+E_{u}+3T_{1u}+T_{2u}+T_{2g}$$

Among these, only the triply degenerate $T_{2g}$ mode is Raman-active and involves relative motion of the oxygen sublattices, while the copper sublattice remains effectively stationary. The Raman band near ∼143 cm^−1^ is assigned to the $\Gamma_{15}^{-}$ (triply degenerate longitudinal optical, LO) mode of Cu_2_O nanoparticles, whereas the weaker feature at ∼225 cm^−1^ corresponds to the second-order vibration. The relatively low intensity of these modes is consistent with a minor Cu_2_O contribution.

Furthermore, a broad Raman band centered around ∼475 cm^−1^ is attributed to the amorphous glass substrate, arising primarily from bending (δ) and stretching (ν) vibrations of Si–O–Si linkages within the disordered silicate network [57]. Finally, a broad feature near 1100 cm^−1^ is observed, which may arise from C–C stretching vibrations of surface-adsorbed carbon species [28].

## 4. Conclusions

The present study demonstrates that carrier-gas flow rate and deposition time strongly influence the agglomerate morphology, size distribution, and surface accumulation of spark-ablated Cu nanoparticles. SEM analysis revealed a transition from compact, near-isotropic agglomerates at low flow rates to elongated, chain-like structures at higher flow rates, reflecting the shift from coalescence- and restructuring-favored conditions toward increasingly kinetically constrained aggregation. Extending deposition times increased surface coverage and agglomerate overlap while preserving the underlying morphological motifs. TEM analysis indicated predominantly spherical nanoparticles with a lognormal size distribution centered at ∼10 nm. Diffraction-based structural analysis confirmed the presence of oxide phases; however, unambiguous identification was not possible due to substrate contributions and peak overlap. In contrast, Raman spectroscopy indicated CuO as the dominant oxide phase with minor Cu_2_O contributions; the present measurements do not resolve the relative contributions of in-flight and post-deposition oxidation. This distinction highlights the complementary roles of diffraction and spectroscopic techniques, where substrate-dominated SAED measurements contrast with the phase-selective sensitivity of Raman spectroscopy.

Comparison with our previous work on Bi–Te nanoparticles further indicates that the detailed agglomeration response is material-dependent, highlighting the importance of accounting for material-specific properties when interpreting spark-ablation processing trends.

Overall, this study establishes clear processing–structure relationships between carrier-gas flow rate, deposition time, and the resulting agglomerate morphology of spark-ablated Cu nanoparticles, while highlighting the complementary roles of diffraction and Raman spectroscopy in assessing oxide phase formation following ambient exposure. These results provide a basis for further optimization of spark-ablation processing conditions for Cu-based nanostructured materials.

## Figures and Tables

**Table 1:** Summary of deposition parameters for all Cu nanoparticle samples prepared by spark ablation, showing flow rate and deposition time for the two experimental series.

Sample ID	Flow Rate (L min^−1^)	Time (h)
Cu-0.5L-2h	0.5	2
Cu-1.5L-2h	1.5	2
Cu-2.5L-2h	2.5	2
Cu-3.5L-2h	3.5	2
Cu-0.5L-5h	0.5	5
Cu-1.5L-5h	1.5	5

**Table 2:** Average spatial frequencies and calculated lattice spacings from FFT analysis of HRTEM images. Each value represents the mean of multiple measurements per point.

Point	Lattice Spacing (Å)	CuO-Consistent (hkl)
1	2.27 ± 0.06	(111), (200)
2	1.60 ± 0.02	(021), (202)
3	1.38 ± 0.03	(022), (113), ($\bar{2}$21)
4	1.15 ± 0.02	(222), (312), (400)
5	0.97 ± 0.02	(402), ($\bar{1}$15), ($\bar{2}$24), ($\bar{4}$21), (420)
6	0.82 ± 0.01	(042)

**Table 3:** Observed SAED d-spacing ranges and corresponding phase assignments based on comparison with reference values for Si and Cu-containing phases. The majority of observed rings correspond to Si substrate reflections, while features in the 2.03–2.14 Å range indicate contributions from Cu-containing phases (metallic Cu and/or Cu_2_O), which are not uniquely assignable due to peak overlap.

Observed *d* (Å)	Reference (hkl)	Interpretation
3.10–3.23	Si (111) 3.14	Si (dominant)
2.03–2.14	Cu (111) 2.09/Cu_2_O (200) 2.13	Cu-containing phase (non-unique)
1.92–1.97	Si (220) 1.92	Si-dominated, non-unique
1.63–1.66	Si (311) 1.64	Si-dominated, non-unique
1.35–1.36	Si (400) 1.36	Si
1.25	Si (331) 1.25	Si

## Data Availability

The data supporting the findings of this study, including SEM and TEM images, Raman spectra, nanoparticle size distribution analyses, and additional FFT and SAED datasets, are available within the article and its Supplementary Information. Raw datasets are available from the corresponding author upon reasonable request.

## Supplementary Materials

The following supporting information can be downloaded at https://www.mdpi.com/article/10.3390/nano16191270/s1: Figure S1: Additional SAED pattern acquired from a different region, showing concentric diffraction rings dominated by Si reflections; Figure S2: Additional SAED pattern confirming Si-dominated diffraction rings with weak and non-unique contributions from Cu-containing phases; Figure S3: Additional SAED pattern showing similar ring structures, confirming the reproducibility of the substrate-dominated diffraction response; Table S1: Measured spatial frequencies and calculated lattice spacings extracted from SAED patterns across multiple regions; Figure S4: FFT pattern extracted from an additional HRTEM region, showing concentric rings corresponding to nanocrystalline domains; Figure S5: FFT pattern from a different region, confirming reproducibility of the spatial-frequency distribution; Figure S6: FFT pattern from an additional region, further confirming consistency across multiple sampling locations; Table S2: Additional lattice spacings extracted from FFT analysis of multiple HRTEM regions; Figure S7: Raman spectrum of the bare glass substrate used for nanoparticle deposition, showing the characteristic broad substrate-related Raman contribution; Figure S8: Comparison of Raman spectra acquired from the deposited sample, bare glass substrate, and CuO powder under comparable measurement conditions; the spectra are vertically offset for clarity; Figure S9: Additional SEM micrographs acquired from multiple substrate regions at carrier-gas flow rates of 0.5 L min^−1^ (a–e), 1.5 L min^−1^ (f–j), 2.5 L min^−1^ (k–o), and 3.5 L min^−1^ (p–t). For each condition, the first panel is a low-magnification overview showing the distribution of deposited features, followed by four higher-magnification images showing individual deposited structures. The images document smaller isolated features and the recurrence of compact, irregular, and elongated micrometre-scale structures. Smooth spherical features visible in some fields may include a contribution from spark-generated droplets.
