# Supplementary material for: Flow-Rate-Controlled Agglomeration and Ambient Oxide Phase Characteristics of Spark-Ablated Copper Nanoparticles
Source: Nanomaterials (Basel). 2026 Oct 8;16(19):1270. doi: 10.3390/nano16191270 (PMC13667034; doi:10.3390/nano16191270)
Supplement: Supplementary file 1 [file nanomaterials-16-01270-s001.zip › nanomaterials-4538594-supplementary.pdf]

Supplementary Information

Flow-Rate-Controlled Agglomeration and Ambient Oxide Phase Characteristics of Spark-Ablated Copper Nanoparticles

Joumana El-Rifai et al.

S1. Additional SAED Analysis

To assess the reproducibility of the diffraction features discussed in the main text, selected area electron diffraction (SAED) patterns were acquired from multiple regions across the sample. The additional SAED patterns confirm that the diffraction response is consistently dominated by reflections originating from the Si substrate, with only limited contributions from Cu-containing phases.

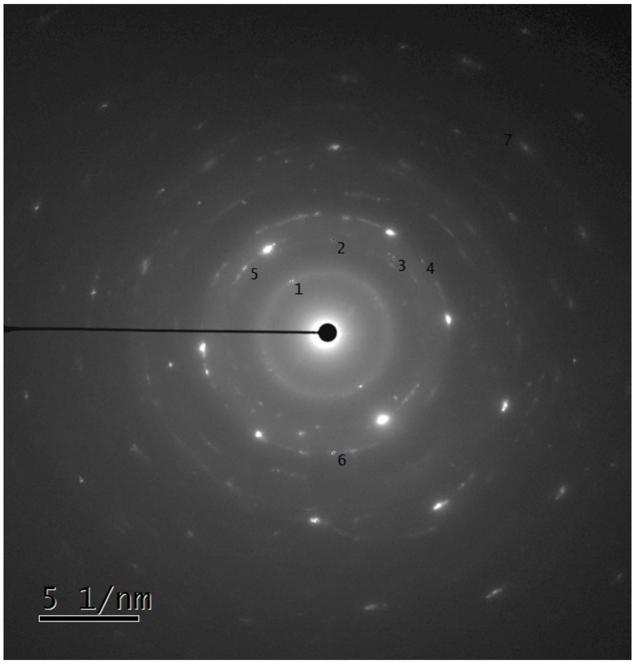

Figure S1: Additional SAED pattern acquired from a different region, showing concentric diffraction rings dominated by Si reflections.

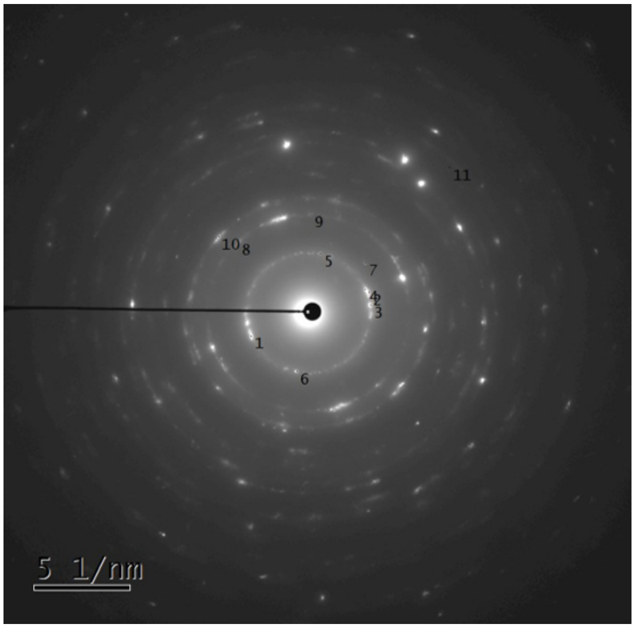

Figure S2: Additional SAED pattern confirming Si-dominated diffraction rings with weak and non-unique contributions from Cu-containing phases.

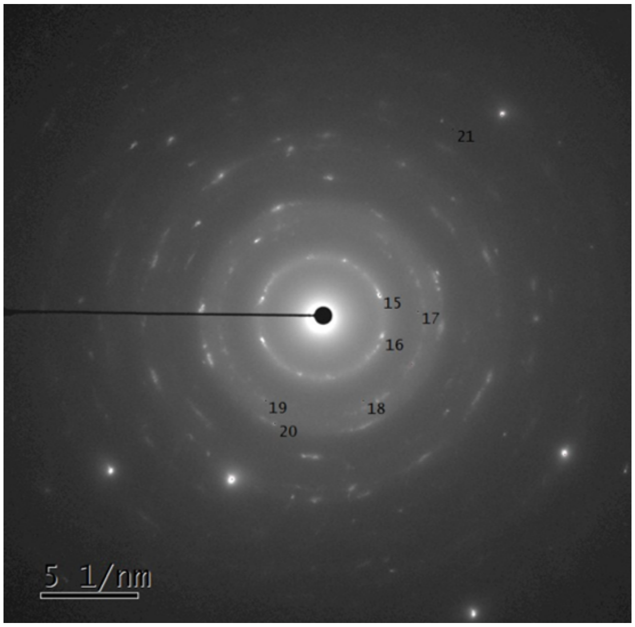

Figure S3: Additional SAED pattern showing similar ring structures, confirming the reproducibility of the substrate-dominated diffraction response.

S2. Extracted d-spacing values

The d-spacing values were extracted from radial intensity profiles of the SAED patterns using ImageJ. Table S1 reports the measured spatial frequencies and corresponding lattice

spacings for all analyzed diffraction features. Larger d-spacing values ($\geq$ 4 Å) arise from low-frequency components and background contributions in the radial intensity profiles and are not considered for phase identification.

Table S1: Measured spatial frequencies and calculated lattice spacings extracted from SAED patterns across multiple regions.

Peak index	Spatial frequency (nm^{-1})	Lattice spacing (Å)
1	3.094	3.232
2	4.710	2.123
3	5.136	1.947
4	6.124	1.633
5	5.165	1.936
6	6.020	1.661
7	13.441	0.744
8	3.162	3.163
9	3.939	2.539
10	5.086	1.966
11	5.066	1.974
12	6.050	1.653
13	10.536	0.949
14	3.220	3.106
15	4.914	2.035
16	4.792	2.087
17	5.208	1.920
18	6.017	1.662
19	11.714	0.854

S3. Additional FFT Analysis

To evaluate the reproducibility of the local structural features identified in the main text, additional fast Fourier transform (FFT) analyses were performed on multiple regions of the HRTEM images (Fig.S4–S6). The extracted lattice spacings are summarized in Table S2.

The additional FFT measurements show consistent clustering of lattice spacings around ~2.3 Å, ~1.6 Å, ~1.38 Å, ~1.16 Å, and ~0.97 Å, in agreement with the values reported in the main text.

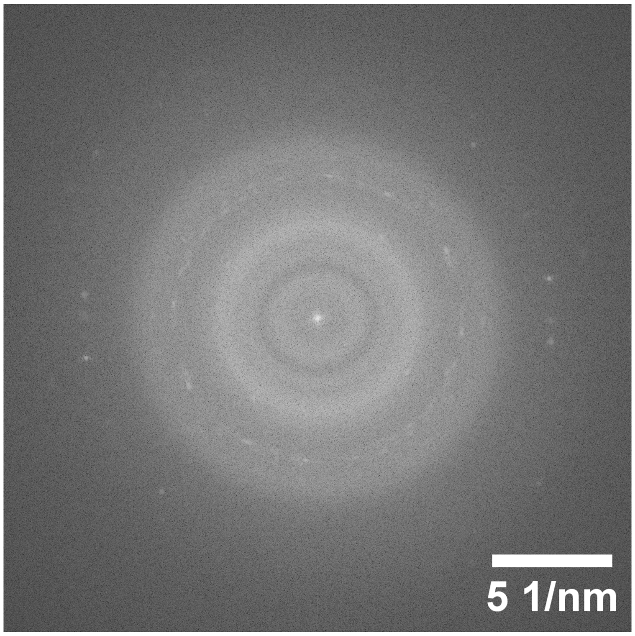

Figure S4: FFT pattern extracted from an additional HRTEM region, showing concentric rings corresponding to nanocrystalline domains.

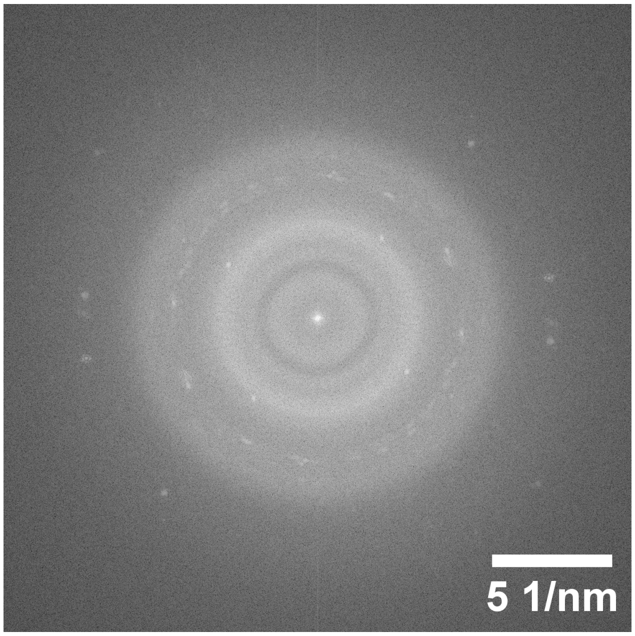

Figure S5: FFT pattern from a different region , confirming reproducibility of the spatial frequency distribution.

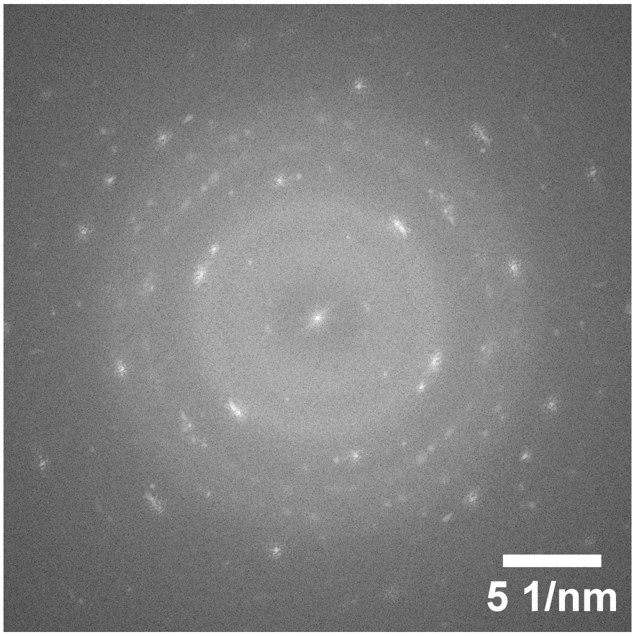

Figure S6: FFT pattern from an additional region, further confirming consistency across multiple sampling locations.

Table S2: Additional lattice spacings extracted from FFT analysis of multiple HRTEM regions (Fig.S4–S6).

Index	Lattice spacing (Å)
1	2.325
2	1.696
3	2.291
4	2.306
5	2.340
6	1.647
7	1.642
8	1.029
9	3.871
10	2.248
11	2.248
12	1.591
13	1.573
14	1.563
15	1.384
16	1.161
17	0.967
18	0.949
19	0.791

S4. Additional Raman Analysis

Raman measurements were performed at different laser powers and at multiple locations on the deposited material to assess signal stability, spatial reproducibility, and the possibility of laser-induced sample modification. The characteristic Raman features were preserved across the investigated laser-power range, with no additional bands or systematic spectral changes indicative of laser-induced phase transformation (Fig. S7).

Reference Raman spectra were also acquired from the bare glass substrate and CuO powder under comparable measurement conditions. Comparison with the deposited sample helps distinguish the broad contribution of the glass substrate from the characteristic CuO-related Raman features observed in the deposited material (Fig. S8).

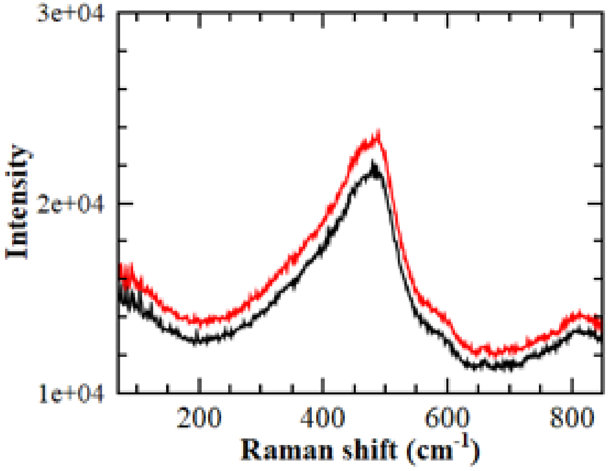

Figure S7: Raman spectrum of the bare glass substrate used for nanoparticle deposition, showing the characteristic broad substrate-related Raman contribution.

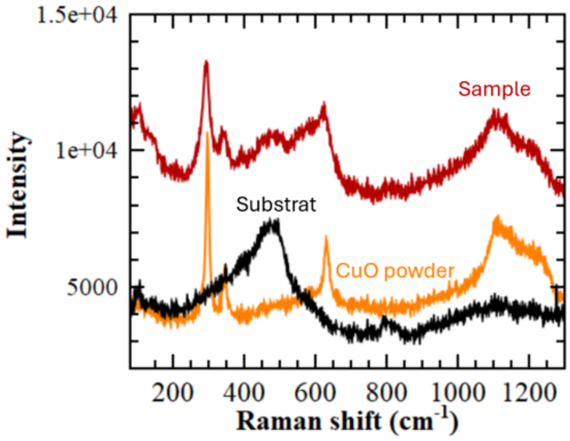

Figure S8: Comparison of Raman spectra acquired from the deposited sample, bare glass substrate, and CuO powder under comparable measurement conditions. The spectra are vertically offset for clarity.

S5. Additional SEM Analysis

To further document the representativeness of the deposited morphologies, additional SEM micrographs were examined from multiple substrate regions across all four carrier-gas flow rates. The image set includes large-area views showing the distribution of deposited features across the substrate and higher-magnification views illustrating the recurring compact, irregular, and elongated structures. Smaller isolated deposited features are also visible in the large-area images.

These micrographs characterize the deposited material following aerosol transport and diffusion-based collection and should not be interpreted as a direct measurement of the gas-phase aerosol size distribution at the spark-generator outlet. At the available SEM resolution, the individual approximately 10 nm primary particles within the micrometer-scale deposits cannot be resolved; their size is determined independently by TEM.

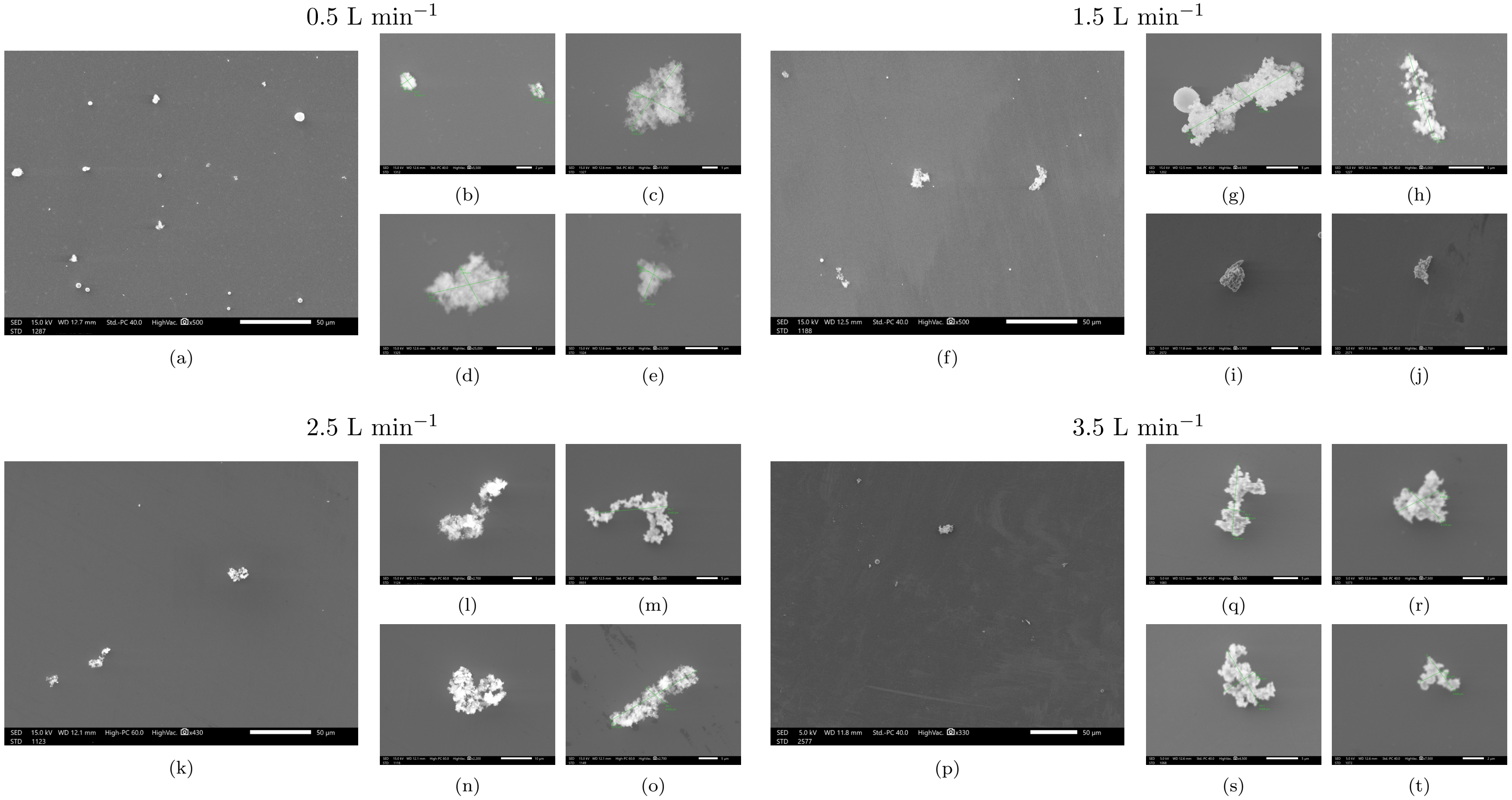

Figure S9: Additional SEM micrographs acquired from multiple substrate regions at carrier-gas flow rates of 0.5 L min^{-1} (a–e), 1.5 L min^{-1} (f–j), 2.5 L min^{-1} (k–o), and 3.5 L min^{-1} (p–t). For each condition, the first panel is a low-magnification overview showing the distribution of deposited features, followed by four higher-magnification images showing individual deposited structures. The images document smaller isolated features and the recurrence of compact, irregular, and elongated micrometre-scale structures. Smooth spherical features visible in some fields may include a contribution from spark-generated droplets.
